# Responsive Polymer Brush Design and Emerging Applications for Nanotheranostics

**DOI:** 10.1002/adhm.202000953

**Published:** 2020-09-06

**Authors:** Danyang Li, Lizhou Xu, Jing Wang, Julien E. Gautrot

**Affiliations:** ^1^ School of Cancer and Pharmaceutical Sciences King's College London 150 Stamford Street London SE1 9NH UK; ^2^ Institute of Bioengineering Queen Mary University of London Mile End Road London E1 4NS UK; ^3^ School of Engineering and Materials Science Queen Mary University of London Mile End Road London E1 4NS UK; ^4^ Department of Materials Imperial College London London SW7 2AZ UK; ^5^ School of Life Sciences Northwestern Polytechnical University Xi'an 710072 China

**Keywords:** biosensing, diagnosis, drug delivery, nanomaterials, nanotheranostics, polymer brushes

## Abstract

Responsive polymer brushes are a category of polymer brushes that are capable of conformational and chemical changes in response to external stimuli. They offer unique opportunities for the control of bio−nano interactions due to the precise control of chemical and structural parameters such as the brush thickness, density, chemistry, and architecture. The design of responsive brushes at the surface of nanomaterials for theranostic applications has developed rapidly. These coatings can be generated from a very broad range of nanomaterials, without compromising their physical, photophysical, and imaging properties. Although the use of responsive brushes for nanotheranostic remains in its early stages, in this review, the aim is to present how the systems developed to date can be combined to control sensing, imaging, and controlled delivery of therapeutics. The recent developments for such design and associated methods for the synthesis of responsive brushes are discussed. The responsive behaviors of homo polymer brushes and brushes with more complex architectures are briefly reviewed, before the applications of responsive brushes as smart delivery systems are discussed. Finally, the recent work is summarized on the use of responsive polymer brushes as novel biosensors and diagnostic tools for the detection of analytes and biomarkers.

## Graphical abstract

Responsive polymer brushes offer unprecedented control of the coating physico‐chemistry and molecular architecture for the design of nanomaterials for the delivery of therapeutics and their simultaneous imaging and biomonitoring. Polymer brushes with precisely controlled responsive and bioactive properties are attractive candidates for the design of the next generation of nanotheranostic platforms.

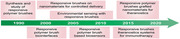

## 1 Introduction

Nanotheranostics integrate diagnostic and therapeutic functions in one system and have received significant attention in the past few decades for the improvement of diagnosis, and the prevention and treatment of diseases.^[^ ^1^, ^2^ ^]^ Advances in nanotheranostics benefit greatly from deeper understanding of the interactions between nanomaterials and biological systems, the refinement of multifunctional nanohybrids for simultaneous diagnosis and therapy, and the ability to harness the unique physicochemical properties of nanomaterials for specific and selective detection and treatment of diseases.^[^ ^3^ ^]^ Although having shown promising results in many in vitro and in vivo studies, the concept of nanotheranostics remains a new paradigm and its clinical use is still in its infancy. In addition to challenges in commercialization, one of the key issues related to the translation of nanotheranostics remains the control and understanding of nano‐bio interactions.^[^ ^2^ ^]^ Upon interaction with biological systems, the physiological properties of nanoparticles determine their stability, pharmacokinetics, biodistribution, and toxicity profiles.^[^ ^4^ ^]^ Those are crucial parameters for assessing their biocompatibility and avoid any adverse immunoreaction or inflammation,^[^ ^5^ ^]^ but also improve efficacy as diagnostic and therapeutic tools. Hence, different techniques and modification strategies of nanomaterials have been developed and characterized to overcome these limitations. Historically, the decoration of nanomaterials with polymers has been particularly successful to tailor and design the properties of these systems.^[^ ^6^ ^]^ This includes self‐assembly of monolayers,^[^ ^7^ ^]^ the stabilization of nanoparticles via ligand exchange methods,^[^ ^8^ ^]^ and the coating with polyelectrolyte assemblies.^[^ ^9^ ^]^ These strategies have enabled the improved dispersity, prolonged circulation time via PEGylation,^[^ ^10^ ^]^ enhanced cellular uptake and loading efficiencies of the therapeutics,^[^ ^11^ ^]^ and increased availability to link with other biomolecules for targeting^[^ ^12^ ^]^ or other purposes.

In particular, polymer brushes, defined as thin polymer coatings in which individual polymer chains are tethered by one chain end to a solid interface, are considered among the most powerful tools to control interface properties. Polymer brushes generated via the “grafting from” approach, in which initiating moieties are coupled to surface and allow the growth of polymer chains, are extremely attractive for the precise design of biomaterials and control over bio‐nano interactions. A number of controlled/“living” polymerization techniques, in particular those based on radical chemistry have been applied to generate such coatings on various types of substrates.^[^ ^13^ ^]^ It enables the grafting density, the thickness, and the chemistry of the coating to be manipulated very readily without altering the bulk mechanical properties of biomaterials.^[^ ^14^ ^]^

**Figure 1:**
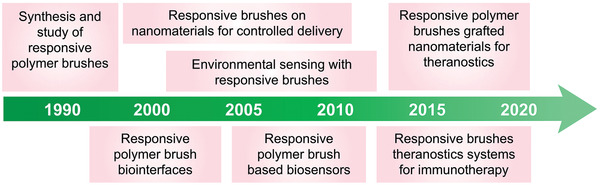
Timeline of major developments of responsive polymer brushes toward nanotheranostics applications.

Stimuli‐responsive polymer brushes exhibit interesting physicochemical and structural changes upon external stimulation. A wide range of stimuli‐responsive polymer materials has been developed, including based on monolayers, multilayer assemblies, gels, and often combined with other nanomaterials and nanoparticles.^[^ ^15^, ^16^ ^]^ Polymer brushes allow an exquisite control over surface properties and real‐time monitoring of biomolecule–surface interactions, which are of great interests in the design of smart biomaterials for nanotheranostics.^[^ ^17^ ^]^ A timeline presenting major developments in the field of responsive polymer brushes toward nanotheranostics applications is detailed in **Figure** **1**. Such thin brush coatings are capable of generating a rapid response without significant alteration of physical properties of the cores/substrates, compared to bulk polymers, which often result in long response times. These features make these coatings particularly attractive for the design of diagnostic and detection tools. Responsive polymer brushes also display highly stable retention and loading of therapeutics such as small nucleic acids owing to the unique crowding architecture of the polymer chains.^[^ ^18^ ^]^ The amount of delivery agents required to achieve therapeutic efficacy could potentially be reduced, leading to safer and more efficient transportation.^[^ ^19^ ^]^ Additionally, the versatile chemistries and functionalities offered during polymer brush synthesis allow the incorporation of multiple stimuli‐responsive moieties. This can also allow the synthesis of two or more chemically different species in one system, i.e., multiresponsive polymer brushes with various architectures, which greatly expand the application scope of responsive polymer brush coatings in theranostic systems.

**Figure 2:**
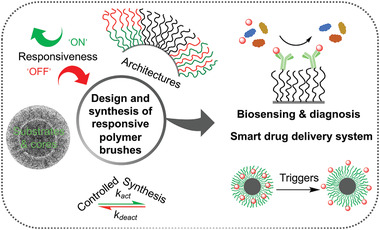
The design and synthesis of responsive polymer brushes and their applications in nanotheranostics. Responsive polymer brush‐based materials can be designed and synthesized via surface‐initiated controlled radical polymerization initiated from various substrates and cores with desired responsiveness and architecture for the applications in biosensing, diagnosis and as smart drug delivery systems. (cryoTEM image of a brush‐coated nanoparticle. Reproduced with permission.^[^ ^23^ ^]^ Copyright 2015, American Chemical Society.)

This review concentrates on the state of the art of responsive polymer brushes that are capable of conformational and chemical changes caused by the external stimuli for nanotheranostic applications (**Figure** **2**). The following sections will discuss the design and synthesis of responsive polymer brushes, discussing some of the synthetic strategies developed from a range of substrates and nanoparticles, the responsive behavior of responsive homo polymer brushes and brushes with multiresponsiveness and multicomponent architectures. We will then focus on the use of responsive polymer brushes as smart delivery systems. Finally, the application of responsive polymer brushes as novel biosensors and diagnostic tools for detection of different analytes and biomarkers will be discussed. For a broader review of polymer brushes in the biomedical field and comparison with other functionalization strategies and responsive polymers, we refer the reader to more exhaustive review articles.^[^ ^14^, ^15^, ^20^, ^21^, ^22^ ^]^

## 2 Design and Synthesis of Responsive Polymer Brushes

### 2.1 Synthetic Strategies toward Polymer Brushes

**Figure 3:**
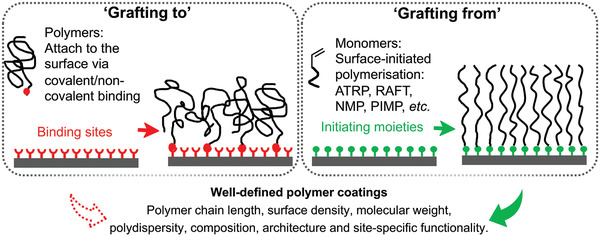
Synthetic approaches for polymer brushes. Polymer brushes can be synthesized on surfaces via “grafting to” and “grafting from” methods.

Surface modification with polymer brushes has given rise to great advances in surface and interface engineering. There are two main strategies for the preparation of polymer brushes, namely, “grafting to” and “grafting from” methods as shown in **Figure** **3**.^[^ ^24^ ^]^ The “grafting to” strategy involves attaching previously synthesized polymers to a substrate or core via covalent bond or noncovalent physical adsorption. A range of polymerization techniques, including cationic, anionic, living free radical, and ring‐opening metathesis polymerization allows the preparation of polymer chains with controlled end functionalities (e.g., amino, hydroxyl, carboxyl, thiol, silane, etc.) that enable tethering to various surfaces. These polymers can be synthesized with narrow molecular weight distributions, and can readily be applied to large area surfaces. Additionally, the grafted polymers can be thoroughly characterized by traditional methods in solutions prior to coating, and many commercially available polymers can be used. However, it is difficult to produce thick and dense polymer brushes due to the steric repulsion between polymer chains and the reaction efficiency between the surface and polymer end‐groups with increasing polymer molecular weight.

In the “grafting from” strategy, polymer brushes are generated in situ from a substrate or core functionalized with initiating moieties. Several surface‐initiated control radical polymerization (SI‐CRP) techniques have been developed, including atom transfer radical polymerization (ATRP), fragmentation chain transfer polymerization (RAFT), nitroxide‐mediated polymerization (NMP), and photoiniferter‐mediated polymerization (PIMP).^[^ ^25^ ^]^ Synthesis and applications of these polymerization techniques have been thoroughly reviewed in the last two decades.^[^ ^14^, ^25^, ^26^ ^]^ These methods rely on establishing a dynamic equilibrium between a low concentration of active propagating chains and a large excess of dormant chains that are unable to terminate via recombination. Among these controlled radical polymerization techniques, ATRP, a typical example of reversible‐deactivation radical polymerization, has been most extensively employed to generate polymer brushes due to its versatile and robust nature.^[^ ^27^ ^]^ It can also tolerate a relatively high degree of impurities, in particular small residual traces of oxygen, which can be removed by oxidation of the ATRP catalyst. Commercially available ready‐to‐use reagents also make ATRP accessible to many laboratories. However, due to the use of metal catalyst, it is challenging to polymerize monomers that can react or complex with the catalyst, such as pyridine‐containing or acidic monomers in a controlled manner. However, Matyjaszewski and co‐workers^[^ ^28^ ^]^ have recently reported a direct polymerization of methacrylic acid through a combination of electrochemically mediated ATRP, supplemental activator, and reducing agent ATRP, yielding high conversions using inexpensive and nontoxic reagents. Several other approaches such as activator (re)generated by electron transfer and initiator for continuous activator regeneration have been employed to reduce the concentration of the copper catalyst that is required for polymerization.^[^ ^29^ ^]^

In contrast, to ATRP, RAFT makes use of a chain transfer agent (RAFT agent), e.g., in the form of a thiocarbonylthio compound, to afford control over the polymerization process. It is relatively simple and versatile, however, comparatively few chain transfer agents are commercially available and some require multistep synthesis.^[^ ^30^ ^]^ NMP is based on the reversible activation/deactivation of growing polymer chains by nitroxide radicals.^[^ ^31^ ^]^ It is a catalyst‐free method which simplifies the process of purification and reduces the chances of introduction of impurities. This is particularly attractive for applications that are sensitive to catalysts involved in ATRP and RAFT. As many surface‐reactive NMP initiators are also not commercially available, additional synthesis may be required. It also limits the range of monomers and surfaces that can be used, because of the relatively high temperature required for polymerization (e.g., at which gold‐thiol bonds will degrade).^[^ ^32^ ^]^ SI‐PIMP is based on the use of iniferters, which act as initiators, transfer agents, and terminators. PIMP is advantageous due to its synthetic simplicity, requiring few reaction components and proceeding via simple application of UV irradiation. It also offers a versatile route to prepare 2D and 3D microstructured polymer brushes without limitation of the types of monomers.^[^ ^33^ ^]^ However, it is challenging to apply SI‐PIMP to photosensitive surfaces and monomers.

The unique characteristic properties of these controlled/“living” radical polymerization (e.g., fast initiation and propagation) offer an unprecedented opportunity for the accurate control polymers brush architectures, with controlled brush thickness, density, molecular weight, composition, and site‐specific functionality. In addition to a wide range of responsive monomers, SI‐CRP also enables the control of more complex architectures of polymer brushes, e.g., block copolymer brushes, mixed brushes as well as gradient brushes. These techniques are well‐suited for the preparation of various functional surfaces and structured biomaterials for applications in nanotheranostics.

### 2.2 Grafting Responsive Polymer Brushes from Various Substrates and Nanoparticles

**Figure 4:**
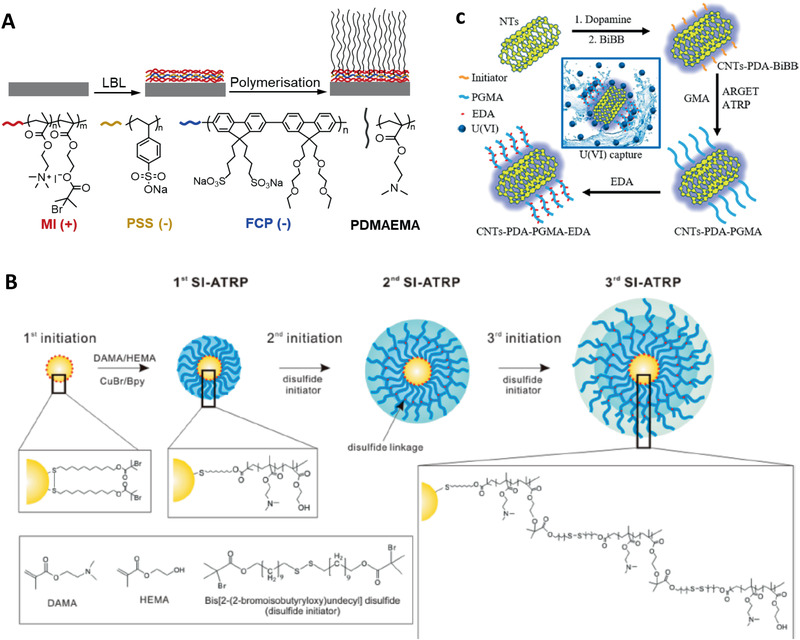
Grafting responsive polymer brushes from various surfaces and cores. A) Core‐independent approach for synthesis of responsive polymer brushes from cationic macroinitiators. Reproduced with permission.^[^ ^39^ ^]^ Copyright 2019, The Royal Society of Chemistry. B) Multishell responsive polymer brushes were initiated from disulfide ATRP initiator‐modified gold nanoparticles. Reproduced with permission.^[^ ^40^ ^]^ Copyright 2018, American Chemical Society. C) Mussel‐chemistry‐inspired approach for coating carbon materials with dense and homogenous responsive polymer brushes. Reproduced with permission.^[^ ^41^, ^42^ ^]^ Copyright 2016, American Chemical Society.

Successful growth of responsive polymer brushes has been applied to a diverse range of surfaces including silicon, silica, gold, metal oxide, carbon‐based surfaces, and semiconductors. For “grafting from” methods, appropriate initiating moieties are required to pre‐modify these surfaces. Tethering a chloro‐ and alkoxy‐silane functionalized initiators to an oxidized silicon substrate is the most frequently applied route for the generation of polymer brushes via SI‐CRP. This strategy is routinely used for the functionalization of a wide range of surfaces, including glass, quartz,^[^ ^34^ ^]^ porous,^[^ ^19^, ^35^ ^]^ and nonporous silica particles,^[^ ^18^, ^36^ ^]^ as well as other nanoparticles displaying a silica shell (e.g., Fe_3_O_4_/silica nanoparticles^[^ ^37^ ^]^). Noncovalent adsorption of polyelectrolyte macroinitiators (e.g., cationic trimethylammonium‐based macroinitiator and anionic sulfate‐based macroinitiator) has also demonstrated the capability of modification of silicon oxide surfaces and efficient initiation polymerization.^[^ ^38^ ^]^ Gautrot et al.^[^ ^39^ ^]^ recently reported a core‐independent approach for synthesizing dense responsive polymer brushes, based on the adsorption of a polyelectrolyte macroinitiator on the surface of nanomaterials with a wide range of core chemistries (**Figure** **4**A). By controlling the deposition of the macroinitiator and polymerization condition, similar kinetics of brush growth were achieved compared with brushes prepared via silane mono‐functional initiators. Additionally, attractive fluorescent conjugated polyelectrolytes with high extinction coefficients and uniquely stable fluorescence were allowed to directly label the responsive polymer brush‐coated nanomaterials when combined to macroinitiators, while retaining the high degree of design freedom of core type and brush chemistry.

The high affinity of thiols and disulfides for gold surface makes it possible to generate well‐defined polymer brushes from gold via SI‐CRP. Choi et al.^[^ ^43^ ^]^ have reported the grafting of thermo‐responsive poly(*N*‐isopropylacrylamide) (PNIPAAm) brushes on gold nanoparticles via a disulfide ATRP initiator. In addition, using disulfide initiators, multiple shells of poly(2‐(dimethylamino) ethyl methacrylate‐2‐hydroxyethyl methacrylate) (DMAEMA‐HEMA) copolymer brushes were prepared on gold nanoparticles using multiple SI‐ATRP processes (Figure 4B).^[^ ^40^ ^]^ Amphiphilic gold nanoparticles with mixed responsive polymer brushes were synthesized via sequential “grafting to” (ligand exchange) and “grafting from” (SI‐ATRP) reactions. Specifically, gold nanoparticles were capped with a binary mixture of methoxy‐poly(ethylene glycol)‐thiol and the ATRP initiator 2,2′‐dithiobis[1‐(2‐bromo‐2 methylpropionyloxy)]ethane (DTBE), via ligand‐exchange reaction and subsequent initiation of a pH‐responsive brush.^[^ ^44^ ^]^

Apart from silicon/silica and gold surfaces, polymer brushes can also be generated from other types of surfaces/cores. For metal oxide surfaces such aluminium, titanium, or iron oxide, one commonly used strategy is the functionalization with triethoxy‐ or trichlorosilane moieties, forming metal‐O‐Si bonds and allowing the growth of polymer brushes. Alternatively, ligand‐exchange reactions can also be introduced to graft ATRP initiators, e.g., to Fe_3_O_4_ nanoparticles stabilized by oleic acid.^[^ ^45^, ^46^ ^]^ In contrast, pristine carbon materials are relatively chemically inert. However, after surface oxidation (typically through treatment in a mixed concentrated acid solution), initiators can be functionalized through esterification with carboxylic and hydroxyl bearing carbon surfaces. Nanodiamonds exhibit stable green fluorescence. Grafting polymer brushes on nanodiamonds can be achieved by reacting a bromide initiator with *N*,*N*′‐carbonyldiimidazole‐activated nanodiamond cores, followed by ATRP on the surface. Similarly, Yan et al.^[^ ^47^, ^48^ ^]^ reported the coating of an ATRP initiator from carbon nanotubes (CNTs), which also involves the oxidation of CNTs, modification with thionyl chloride to form CNT‐COCl, reacting with ethylene glycol to form CNT‐OH prior to reaction with bromide initiator moieties. Such functionalization typically results in limited anchorage of the initiators as it heavily relies on the availability of the functional groups on CNTs. Too high functionalization would result in disruption of the CNT structure and photophysical properties. To address this issue, inspired by the adhesive behavior of mussel proteins, biocompatible and uniform polydopamine (PDA) coating can be assembled at the surface of CNTs by spontaneous self‐polymerization of dopamine under mild alkaline conditions. The high concentration of catechol and amine groups on the PDA surface enables the generation of high density polymer brushes from CNTs surface (Figure 4C).^[^ ^41^, ^42^ ^]^ In contrast, the functionalization of polymer brushes from the surface of semiconductor particles such as quantum dots (QDs) can reduce the density of surface dangling bonds and defect levels, and improve the photoluminescence intensity and stability of the nanocrystals in aqueous media.^[^ ^49^ ^]^ Using this approach, Lai et al.^[^ ^50^ ^]^ reported a novel and versatile method for the preparation of multitype polymer brushes from the surface of QDs, involving functionalization with an azo initiator onto the surface of QDs, followed by reaction with 4,4′‐azobis (4‐cyanovaleric acid) via an ester linkage.

It is worth noting that beyond the control of the grafting density of polymer brushes, the curvature defined by core of the nanomaterial also modulates the responsive behavior of the resulting coating,^[^ ^51^ ^]^ which typically differs from the same free polymers in solution.^[^ ^52^ ^]^ For instance, Klok and co‐workers^[^ ^53^ ^]^ have reported that the lower critical solution temperature (LCST) of poly(poly(ethylene glycol) methacrylate) (PPEGMA) polymers is higher than that of PPEGMA‐grafted gold nanoparticles, although the stability of particles such gold particles should be further explored. In addition, nanoparticles have much larger surface areas than flat substrates, leading to high polymer brush contents, in which the curvature reduces steric hindrance between polymer chains and associated impact on brush growth and physicochemical properties.^[^ ^26^ ^]^ Overall, polymer brushes offer unique opportunities to alter physicochemical properties of nanomaterials without compromising the properties of their core. For example, Fe_3_O_4_ cores retain their magnetic properties and enable the remote activation of thermo‐responsive brushes, for the control of drug release.^[^ ^54^ ^]^ The following section will examine how the engineering of the chemical structure of polymer brushes enables to confer responsiveness to nanomaterials.

### 2.3 Responsive Behaviors of Polymer Brushes

**Figure 5:**
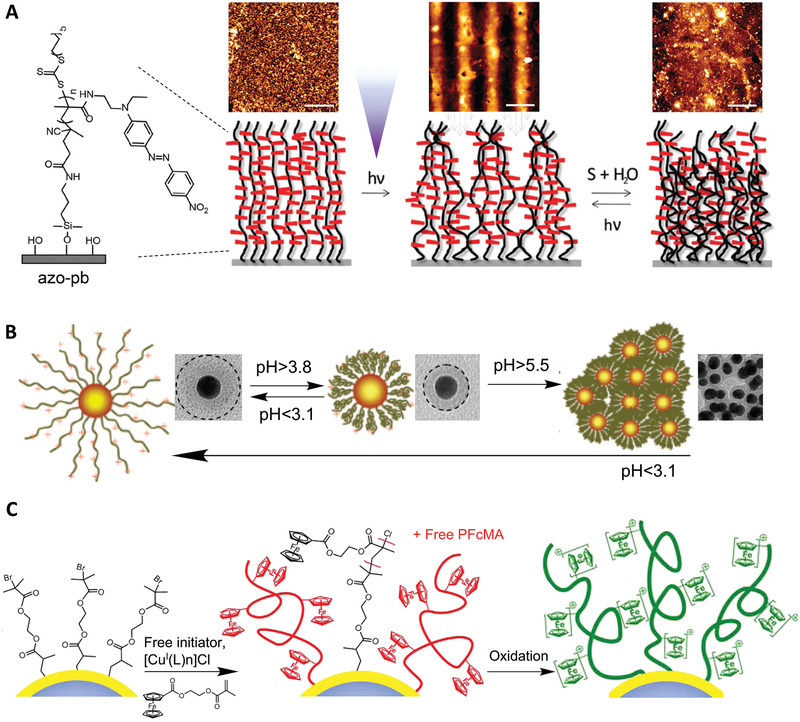
Responsive polymer brushes. A) Topographic changes of photoresponsive polymer brushes. Reproduced with permission.^[^ ^60^ ^]^ Copyright 2017, The Royal Society of Chemistry. B) pH‐responsive P4VP brushes grafted from gold nanoparticles exhibiting a two‐stage response, which affects the aggregation of particles at different pH. Reproduced with permission.^[^ ^67^ ^]^ Copyright 2008, American Chemical Society. C) Reversible redox responsive of poly(2‐(methacryloyloxy)ethyl ferrocenecarboxylate) (PFcMA) brushes coating polystyrene nanoparticles. Reproduced with permission.^[^ ^68^ ^]^ Copyright 2012, American Chemical Society.

The responsive behavior of polymer brushes is largely dependent on the brush chemical structure and architecture (e.g., chemistry, thickness, density). For example, thermo‐responsive PNIPAAm brushes display an LCST at which they undergo a phase transition.^[^ ^55^ ^]^ The phase transition/separation phenomenon of PNIPAAm is fast and reversible.^[^ ^56^ ^]^ Below the LCST, PNIPAAm exhibits a hydrated random coil conformation due to hydrogen bonding between the hydrophilic group and water molecules, whereas with increasing temperatures, hydrophobic interactions between polymer chains dominate, resulting in a collapse and globular conformation.^[^ ^57^ ^]^ Other brushes were designed to display photoresponsiveness, owing to the introduction of photoactive moieties (e.g., azobenzenes or spiropyranes). Under irradiation, these molecules undergo conformational changes, thereby influencing the local chemical environment and brush conformation.^[^ ^58^ ^]^ Flexible linkage of the photosensitive moieties, which can be achieved within polymer brushes, is key to their functionality.^[^ ^59^ ^]^ This can be harnessed to regulate dynamic interactions between cells and the extracellular matrix (ECM) in vitro, as it allows to precisely control the localization of ECM molecules on a substrate and also allows the production of complex patterns (**Figure** **5**A) in one step without any contact or contamination.^[^ ^60^ ^]^ Electrically sensitive polymer brushes, such as polystyrene sulfonate (PSS) brushes displayed conformational changes in response to an applied external field.^[^ ^61^ ^]^ This type of brushes enables the control of physicochemical properties via the magnitude of the current, the duration of the electrical field applied, and the interval between pulses.^[^ ^62^ ^]^ It was demonstrated that polymer brushes grafted from conductive poly(3,4‐ethylenedioxythiophene) (PEDOT) films display dynamic switch of brush conformation, dependent on multiple stimuli including the corresponding electrode potential.^[^ ^63^ ^]^ Travas‐Sejdic et al. have explored the functionalization of conducting polymer brushes in biomedical applications,^[^ ^64^ ^]^ in particular for electrochemically triggered drug delivery^[^ ^65^ ^]^ and DNA sensing.^[^ ^66^ ^]^

In addition to systems physically stimulated, chemically sensitive polymer brushes can change conformation in response to environmental pH, ionic strength, specific electrolytes, and solvent. Polyelectrolyte brushes typically respond to changes in pH, ionic strength, and the type of ionic species in solution via important rearrangement of their conformation.^[^ ^69^ ^]^ For example, quaternary ammonium poly(2‐methacrylolyloxyethyl trimethylammonium chloride) (PMETAC) brushes respond to the presence of percholorate ions, resulting to rapid changes in hydrodynamic diameter of associated nanoparticles and the control of their aggregation.^[^ ^70^ ^]^ Conventional zwitterionic polymers display opposite charges connected to each other via a covalent linkage. Jiang et al.^[^ ^71^ ^]^ have reported the rational design of polymer brushes with tertiary amine groups and carboxylic acid groups, which are capable to reversibly switch between three distinct charged states (cationic, zwitterionic, and anionic), depending on pH. This enables the control of antifouling properties, depending on pH, and the resistance to human serum and plasma protein adsorption in physiological conditions.

In an acidic environment, polyacid brushes^[^ ^72^ ^]^ such as poly(acrylic acid) (PAA) and poly(methacrylic acid) (PMAA) are protonated and hydrophobic, leading to their dehydration and collapse. However, in basic conditions, deprotonated polyacid brushes become negatively charged and will swell due to the associated Coulombic repulsion. The pH‐response of polybase brushes^[^ ^73^ ^]^ (e.g., poly(*N*,*N*′‐dimethylaminoethyl methacrylate)) (PDMAEMA), poly(2‐(diethylamino) ethyl methacrylate) (PDEAEMA), and poly(4‐vinylpyridine) (P4VP)) is opposite to that of polyacid brushes. Their wet thickness decreases with increasing pH due to the deprotonation of the charged side groups. This typically results in the aggregation of particles decorated with such brushes. For instance, P4VP‐grafted gold nanoparticles exhibited a two‐stage pH responsiveness (Figure 5B).^[^ ^67^ ^]^ At low pH (< 3.1), polymer chains are positively charged and hydrated due to the formation of pyridinium ions. Hence, polymer chains extend under the associated electrostatic repulsion, resulting in the excellent dispersion of nanoparticles. At an intermediate pH range of 3.8–4.4, pyridinium groups are gradually deprotonated and water becomes a poor solvent. Therefore, polymer chains are collapsed onto the gold cores, but nanoparticles remain monodisperse, presumably due to residual positive charges at their surface. This response is reversible due to the protonation/deprotonation process of pyridine groups. At pH higher than 5.5, the very low concentration of H^+^ ions results in a drastic decrease in surface charge and the aggregation of nanomaterials. Polyelectrolyte brushes also respond to different ionic strength,^[^ ^74^ ^]^ resulting in a rich variety of conformational regimes.^[^ ^75^, ^76^ ^]^ At low salt concentrations, brushes display low surface charge, so chain extension is very limited. With increasing ionic strength, counterion exchange allowing brushes to charge while simultaneously swelling due to the increased osmotic pressure (osmotic brush regime). At higher salt concentrations, the brushes are fully ionized, but charges are screened, resulting in brush collapse (salted brush regime).^[^ ^77^ ^]^

Redox responsiveness has been extensively explored for biomedical applications, due to the difference in redox environment in the circulation/extracellular fluids and intracellular compartments.^[^ ^78^ ^]^ Redox responsiveness can be achieved via the incorporation of redox‐responsive linkages such as disulfides and diselenides. For instance, magnetic nanoparticles were successfully modified with PPEGMA brushes, which were further conjugated with thiols via disulfide linkages and exhibited glutathione‐dependent responsiveness.^[^ ^79^ ^]^ Another interesting redox‐active couple is the ferrocene/ferrocenium moiety. Mazurowski et al.^[^ ^68^ ^]^ reported the redox responsiveness of poly(2‐(methacryloyloxy)ethyl ferrocenecarboxylate) (PFcMA) brushes on polystyrene nanoparticles with different density, synthesized via ATRP (Figure 5C). Enormous swelling was observed after chemical oxidation of the ferrocene‐containing brush shells, with almost doubling of the hydrodynamic diameter of the shell compared to that prior to oxidation. This responsive behavior was almost fully reversible without any degradation of PFcMA, offering potential design for biosensors.

### 2.4 Multistimuli‐Responsive Polymer Brushes and Multitopography Brushes

The combination of two or more stimuli‐responsive moieties into one system can lead to a more pronounced or more complex response toward the impact of external stimuli.^[^ ^80^, ^81^ ^]^ Some homopolymer brushes naturally exhibit dual responsiveness, such as PDMAEMA brushes (thermo and pH responsive), PNIPAAm brush (thermo and redox responsive), and weak polyelectrolyte brushes (pH and ionic strength responsive). The introduction of multistimuli responsive systems may allow the improvement of the control of physicochemical changes (e.g., hydrophilic–hydrophobic transition), the widening of the switching window, or even change the switching conditions due to the higher level of complexity local physicochemical properties involved. Moreover, the introduction of different topologies can also have an important impact on their responsiveness to external stimuli.

**Figure 6:**
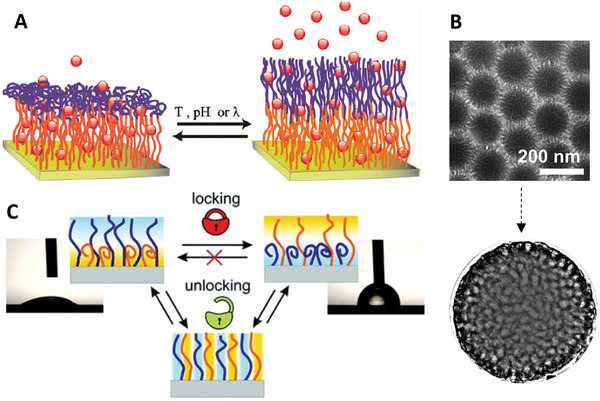
Multistimuli and multitopology polymer brush systems. A) Triple‐responsive block copolymer brushes were designed for controlled opening and closure of brushes in water, enabling the capture and release of a model dye. Reproduced with permission.^[^ ^82^ ^]^ Copyright 2011, American Chemical Society. B) TEM study of the phase morphologies of solvent‐responsive mixed brushes on silica nanoparticles. Reproduced with permission.^[^ ^83^ ^]^ Copyright 2008, American Chemical Society. C) Solvent‐triggered locking/unlocking behaviors of PS‐PAA mixed polymer brushes. Reproduced with permission.^[^ ^84^ ^]^ Copyright 2012, American Chemical Society.

Unlike homopolymer brushes, block copolymer brushes demonstrate a rich phase behavior, which is based on the segregation of different blocks, e.g., displaying different solvent affinities. Surface properties of ABC triblock copolymer poly‐(dimethylsiloxane)‐block‐polystyrene‐block‐poly(dimethylsiloxane) (PDMS‐*b*‐PS‐*b*‐PDMSA) synthesized on silicon wafer could be reversibly controlled to present either PS or PDMS segments by the treatment with toluene, or a mixture of toluene/hexane.^[^ ^85^ ^]^ Random copolymer brushes consisting of DMAEMA and 10‐(2‐acryloxyethyl)‐30,30‐dimethyl‐6‐nitrospiro‐(2*H*‐1‐benzopyran‐2,20‐indoline) (SPMA) grafted silica nanoparticles displayed triple responsive behaviors including thermo‐, pH‐, and photo‐sensitivity.^[^ ^86^ ^]^ In other systems, the controlled release of biomolecules was achieved utilizing the specific responsiveness of each block. Kumar et al.^[^ ^82^ ^]^ presented a general approach (**Figure** **6**A) based on ATRP to grow sequentially a first block as an inner reservoir for loading a model dye and a second block as a stimuli‐responsive outer layer for controlling the opening and closure of brushes in aqueous conditions. The inner block can either be hydrophobic or hydrophilic depending on the guest molecules, and the switchable outer block can also be designed in response to a temperature or pH change, or to exposure to UV light. This versatile platform is of interest as delivery system for the controlled release of therapeutic molecules.

Much like block copolymers, in mixed polymer brushes, at least two chemically distinct polymers are randomly or alternately grafted to the same substrate/core, displaying unique responsive nanostructures interfaces. The responsiveness of mixed brushes grafted from particles stems from the capacity of two immiscible components to undergo structural re‐organization in a confined geometry in response to environmental variations, as a result of surface energy minimization.^[^ ^87^ ^]^ Thus, the grafted mixed polymer brushes are not necessarily stimuli‐responsive in a conventional sense. Indeed, the use of stimuli‐responsive polymer brushes for designing and synthesizing mixed brush‐grafted particles would further enhance the control of phase segregation, leading to the switching of properties (or functional groups) of the constituent polymers that interact dynamically with their environment.^[^ ^88^ ^]^ The phase morphologies of mixed brushes‐coated silica nanoparticles were comprehensively studied via transmission electron microscopy (TEM) upon response to different solvents (Figure 6B), in which the sample underwent lateral microphase separation, producing a nearly bicontinuous, random worm‐like pattern.^[^ ^83^ ^]^ Minko et al.^[^ ^89^ ^]^ reported an interesting mixed brush‐coated stimuli‐responsive colloidal system, which enabled the reversible control of interactions between particles, as well as particles and their environment, mediated by a change of solvent and pH. The switching properties of mixed brushes have also been demonstrated in environmentally responsive lithography. A pattern “written” in mixed brushes by UV irradiation through a photomask can be reversibly developed and erased by treatments with different solvents.^[^ ^90^ ^]^ It was also found that PS‐PAA mixed polymer brushes (Figure 6C) revealed interesting solvent‐triggered locking/unlocking behaviors, which are substantially dependent on grafting density, molecular weight, and compatibility of the two distinct grafted polymers.^[^ ^84^ ^]^ This could be particularly useful for applications for the development of smart materials, such as active elements of microfluidic devices and for the development of biosensors.

## 3 Emerging Applications in Nanotheranostics

Responsive polymer brushes have found numerous applications in the design of stimuli‐responsive devices, multifunctional thin films, and surfaces. These intelligent polymer coatings have also been extensively studied in the past decade, for application in the biomedical field.^[^ ^14^ ^]^ In the following section, we will focus on discussing the application of responsive polymer brushes as smart delivery systems, combining bioimaging and biosensing/detection platforms for diagnosis.

### 3.1 Triggered Drug Delivery and Therapy

Since the first report of the concept of stimuli‐responsive polymer‐based drug delivery systems in the late 1970s,^[^ ^91^ ^]^ significant research efforts have focused on the design of stimuli‐responsive materials for drug delivery. Theranostic technologies combine therapeutics delivery with bioimaging or diagnosis in one single system, allowing to gain valuable insight into the mechanisms underlying therapeutic efficacy or lack of response. Nonresponsive polymer brush‐functionalized imaging probes, mostly containing oligo(ethylene glycol)^[^ ^45^, ^92^, ^93^ ^]^ have been widely studied. In this section, we will focus on the applications of responsive polymer brushes as controlled delivery systems, combined with nanomaterials cores enabling bioimaging and monitoring. Responsive polymer brush systems used as drug carriers can selectively release their payloads in response to trigger mechanisms, thereby enabling a more precise control of the dosing of therapeutic agents. For example, PDMAEMA with pendant tertiary amines is a temperature‐ and pH‐ responsive polymer, often used as cationic antibacterial surfaces and nonviral gene carriers for biomedical applications.^[^ ^18^, ^36^, ^94^, ^95^ ^]^ Many other responsive polymer brushes have been developed for use in drug delivery. We have summarized recent advances toward these applications in **Table** **1**. In many cases, the responsive polymer brush is anchored to a substrate or core to form a hybrid system that encapsulates or load therapeutic agents for cellular delivery. Nanomaterials such as gold nanocages, magnetic nanoparticles, silica nanotubes, etc., have been reported for such applications. For example, the high loading level of small RNAs and their stabilization within densely packed PDMAEMA brushes has been recently reported.^[^ ^18^ ^]^

**Table 1:** Application of responsive polymer brushes for triggered therapeutic delivery

Cargo (drug)	Stimuli	Responsive polymer brush^a)^	Core	Ref.
Rh6G dye	Temperature	P(OEGMA‐*co*‐MEO2MA)	SWCNTs	^[^ ^96^ ^]^
Rh6G dye	Temperature	PNIPAAm‐*co*‐PAAm	Au nanocage	^[^ ^97^ ^]^
Rh6G dye	pH	PDMAEMA	MSNs	^[^ ^98^ ^]^
RhB dye	pH	PDEAEMA	MSNs	^[^ ^99^ ^]^
Dye (Calcein)	pH	P4VP	MSNs	^[^ ^100^ ^]^
Diol derivatives	Saccharide and temperature	PNIPAAm‐*co*‐PMAA	AuNSs	^[^ ^101^ ^]^
Doxorubicin	Temperature	PEO‐*b*‐PLL	Magnetic nanoparticles	^[^ ^54^ ^]^
4‐acetamidophenol and Ranitidine	Temperature	PNIPAAm	Micro‐porous PC film	^[^ ^102^ ^]^
Doxorubicin	pH	PDEAEMA‐*b*‐POEGMA	Silica nanotube	^[^ ^103^ ^]^
Doxorubicin	pH	PPEMA	MSNs	^[^ ^104^ ^]^
Doxorubicin, IR825 dye	pH and photothermal	PMABH‐*b*‐POEGMA	MSNs	^[^ ^105^ ^]^
Doxorubicin	pH, UV	PNB‐*b*‐POEG	UCNPs (with phototherapy)	^[^ ^106^ ^]^
Doxorubicin	pH	PAA	UCNPs (with phototherapy)	^[^ ^107^ ^]^
Doxorubicin	pH	PAA	UCNPs (with phototherapy)	^[^ ^108^ ^]^
Doxorubicin	pH	PAA	MSNs (with MRI)	^[^ ^109^ ^]^
Doxorubicin	pH and temperature	PNIPAAm	SPIONs (with MRI)	^[^ ^110^ ^]^
Doxorubicin	pH	PAA	SPIONs (with MRI)	^[^ ^111^ ^]^
Doxorubicin	pH and temperature	PNIPAAm‐*co*‐PMAA	SPIONs (with MRI and photothermal therapy)	^[^ ^112^ ^]^
SN_38_	pH and redox	PSN_38_‐*co*‐P4VP	AuNNPs	^[^ ^113^ ^]^
Oxaliplatin	pH and redox	DiPt‐ASlink‐PEG2k	NL919	^[^ ^114^ ^]^
(Ru(bipy)3				
2+) dye, lysozyme	Temperature	PNIPAAm	Fe_3_O_4_/SiO_2_ NPs	^[^ ^115^ ^]^
Doxorubicin, lysozyme	Temperature	PNIPAAm‐*co*‐PAAm	Au nanocage	^[^ ^116^ ^]^
plasmid DNA	pH and temperature	PDMAEMA	SPIONs	^[^ ^117^ ^]^
plasmid DNA	pH	PDMAEMA	SPIONs	^[^ ^118^ ^]^
CPT, plasmid DNA	pH	PDMAEMA	GO	^[^ ^119^ ^]^
plasmid DNA	pH	PDMAEMA	LDHs	^[^ ^120^ ^]^
plasmid DNA	pH	PDMAEMA	Chiral Si nanorods	^[^ ^121^ ^]^
plasmid DNA	Shielding effect	PDMAEMA	Spindly CNCs	^[^ ^122^ ^]^
siRNA	Bioreductivity	Poly(DAMA‐HEMA)	Gold NPs	^[^ ^40^ ^]^
plasmid DNA	pH	PDMAEMA	Silica NPs	^[^ ^36^ ^]^
plasmid DNA, siRNA	pH	PDMAEMA	Silica NPs	^[^ ^95^ ^]^
siRNA	pH	PDMAEMA	Silica NPs	^[^ ^18^ ^]^
siRNA	pH	PDMAEMA	Silica NPs	^[^ ^39^ ^]^
plasmid DNA	pH	PDMAEMA	Nanodiamond (with bioimaging)	^[^ ^123^ ^]^
siRNA	pH	PDMAEMA	UCNPs (with phototherapy)	^[^ ^124^ ^]^
siPD‐L1	Redox	SPDP	Magnetic nanoparticle (MRI imaging)	^[^ ^125^ ^]^
siPD‐L1	Redox	FA‐PEG‐SS‐PEI	SPION (MRI imaging)	^[^ ^126^ ^]^

^a)^ Abbreviations: Rh6G, Rhodamine 6G; RhB, Rhodamine B; SN_38_, 7‐ethyl‐10‐hydroxycamptothecin; CPT, 10‐hydroxycamptothecin; P(OEGMA‐*co*‐MEO2MA), poly(oligo(ethylene glycol) methacrylate‐*co*‐2‐(2′‐methoxyethoxy)ethyl methacrylate); PNIPAAm‐*co*‐PAAm, poly(*N*‐isopropylacrylamide‐*co*‐acrylamide); PDMAEMA, poly(*N*,*N*′‐dimethylaminoethyl methacrylate); PDEAEMA, poly(2‐(diethylamino) ethyl methacrylate); P4VP, poly(4‐vinyl pyridine); PNIPAAm‐*co*‐PMAA, poly[(*N*‐isopropylacrylamide)‐*co*‐(methacrylic acid)]; PSN_38_‐*co*‐P4VP, poly(SN_38_ (7‐ethyl‐10‐hydroxycamptothecin)‐*co*‐4‐vinylpyridine); DiPt‐ASlink‐PEG2k, diisopropyltryptamine‐PEGylated 2‐propionic‐3‐methylmaleic anhydride; PEO‐*b*‐PLL, polyethylene oxide‐*b*‐poly(L‐lysine); PDEAEMA‐*b*‐POEGMA, poly(2‐(diethylamino)ethyl methacrylate)‐*b*‐poly(oligo(ethylene glycol) meth‐acrylate); PPEMA, poly(2‐(pentamethyleneimino)ethyl methacrylate); PMABH‐*b*‐POEGMA, poly(methacrylamide *tert*‐butyl carbazate)‐*b*‐poly(oilgo(ethylene glycol) methacrylate); PNB‐*b*‐POEG, poly(4,5‐dimethoxy‐2‐nitrobenzyl methacrylate)‐*b*‐poly(methoxy polyethylene glycol monomethacrylate); PAA, poly(acrylic acid); poly(DAMA‐HEMA), poly(2‐(dimethylamino) ethyl methacrylate‐2‐hydroxyethyl methacrylate); SPDP, *N*‐Succinimidyl 3‐[2‐pyridyldithio]‐propionate; FA‐PEG‐SS‐PEI, folic acid‐ poly(ethylene glycol)‐disulfide‐poly(ethylene imine); SWCNTs, single‐walled carbon nanotubes; MSNs, mesoporous silica nanoparticles; AuNSs, gold nanoshells; PC, polycarbonate; UCNPs, upconversion nanoparticles; SPIONs, superparamagnetic iron oxide nanoparticles; AuNNPs, nanogapped gold nanoparticles; NL919, 1‐Cyclohexyl‐2‐(5*H*‐imidazo[5,1‐a]isoindol‐5‐yl)ethanol; GO, graphene oxide; LDHs, layered double hydroxides; CNCs, cellulose nanocrystals; NPs, nanoparticles.

**Figure 7:**
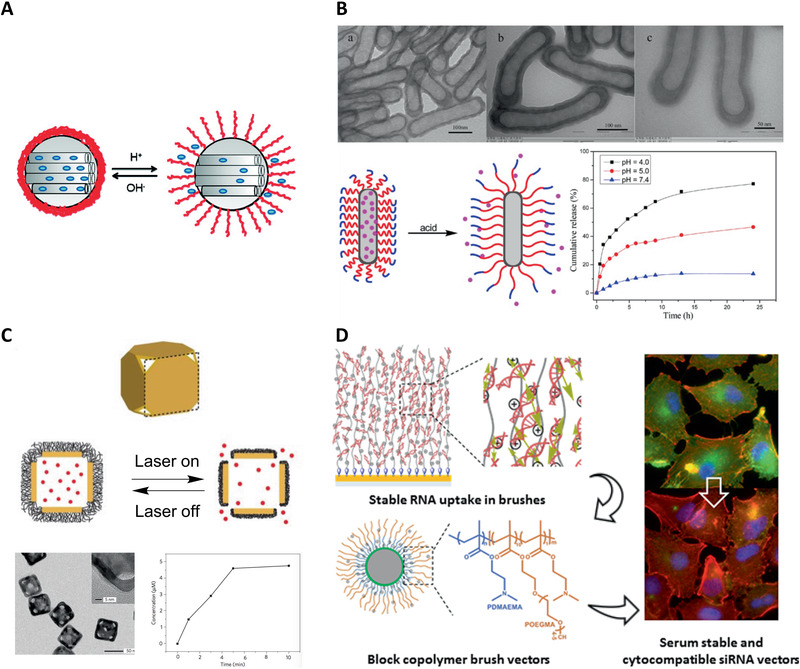
Applications of responsive polymer brushes for triggered drug delivery. A) PDEAEMA‐coated silica nanoparticles and pH‐sensitive controlled release of a model dye. Reproduced with permission.^[^ ^99^ ^]^ Copyright 2010, American Chemical Society. B) Schematic illustration for pH‐responsive diblock copolymers brush grafted silica nanotubes for controlled drug release. Reproduced with permission.^[^ ^103^ ^]^ Copyright 2015, American Chemical Society. C) Gold nanocages coated with responsive polymer brushes for release in response to NIR light. Reproduced with permission.^[^ ^116^ ^]^ Copyright 2009, Springer Nature. D) Highly stable capture of RNAs by dense cationic polymer brushes for the design of cytocompatible, serum‐stable siRNA delivery vectors. Reproduced with permission.^[^ ^18^ ^]^ Copyright 2018, American Chemical Society.

Fluorescent dyes are often used as model molecules for the study of potential drug delivery systems facilitated by responsive polymer brushes. This is due to dyes such as Rhodamine 6G (Rh6G) displaying similar properties compared to therapeutic agents such as doxorubicin (DOX), a common cancer chemotherapy drug, in terms of hydrophobicity, molecular weight (similar size), and surface charge. For instance, thermo‐responsive copolymer brushes poly(oligo(ethylene glycol) methacrylate‐*b*‐poly(diethylene glycol methacrylate) (POEGMA‐*co*‐PMEO2MA) grafted single‐walled carbon nanotubes (SWCNTs) exhibited LCST‐dependent Rh6G loading and release properties.^[^ ^96^ ^]^ During the Rh6G‐loading process, below the LCST, brush chains were extended, thus the Rh6G could easily adsorb within the SWCNT@POEGMA‐*co*‐PMEO2MA. However, the collapsed brush chains can restrict diffusion and retain some of the molecules above the LCST. The thermally controlled drug release was confirmed via UV‐vis spectroscopy. The release rates of Rh6G below the LCSTs were significantly faster than those above the LCST. The pH and temperature responsive nanocarriers were also designed and their efficacy was demonstrated in the case of Rh6G,^[^ ^97^, ^98^ ^]^ Rhodamine B (RhB)^[^ ^99^ ^]^ (**Figure** **7**A), and calcein,^[^ ^100^ ^]^ as summarized in Table 1.

**Figure 8:**
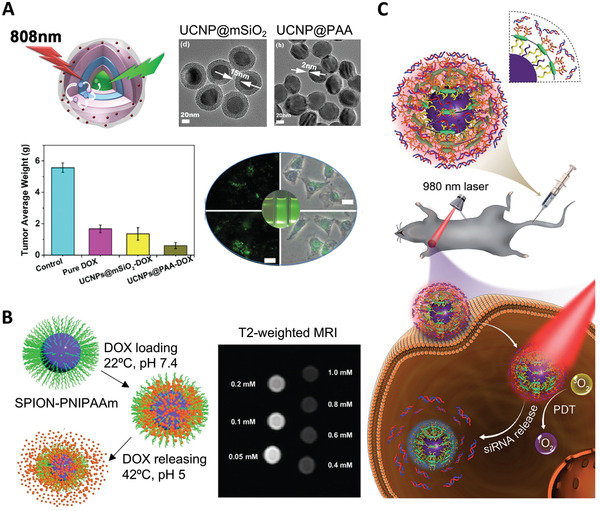
Theranostics design using responsive polymer brushes for dual drug delivery and bioimaging. A) PAA brush‐modified upconversion nanomaterials for highly efficient upconversion luminescence imaging and pH‐responsive drug delivery. Reproduced with permission.^[^ ^108^ ^]^ Copyright 2015, Wiley‐VCH GmbH. B) Dual responsive PNIPAAm brush‐coated SPIONs for controlled doxorubicin delivery and MR imaging. Reproduced with permission.^[^ ^110^ ^]^ Copyright 2017, The Royal Society of Chemistry. C) Schematic representation of photo‐induced charge‐variable nanotherapeutic system for simultaneous photodynamic therapy and siRNA delivery. Reproduced with permission.^[^ ^124^ ^]^ Copyright 2017, Wiley‐VCH GmbH.

The release of small molecule therapeutic agents can be controlled by responsive polymer brush‐based triggered drug delivery systems. DOX is widely used in these studies, owing to its application for chemotherapy. For example, pH‐controlled release of DOX was reported using a biocompatible POEGMA layer grown after a PDEAEMA polymer shell to reduce the toxicity of PDEAEMA‐*b*‐POEGMA copolymer brush‐grafted silica nanotubes (Figure 7B).^[^ ^103^ ^]^ Hybrid responsive polymer brush systems have been explored for imaging‐guided therapy. Near‐infrared (NIR) light‐responsive upconversion nanoparticles (UCNPs) were coated with poly(4,5‐dimethoxy‐2‐nitrobenzyl methacrylate)‐*b*‐poly(methoxy polyethylene glycol monomethacrylate) (PNB‐*b*‐POEG) copolymer brush, in which PNB is a UV‐sensitive hydrophobic polymer and allows the light‐mediated control of drug delivery of DOX.^[^ ^106^ ^]^ Similarly, a multifunctional UCNPs@mSiO_2_‐PAA nanohybrid with UCNPs as core and PAA gated mesoporous silica as shell was reported for controlled release of DOX in vitro.^[^ ^107^ ^]^ In simulated gastric fluid with pH 1.2, DOX molecules were encapsulated in the pores of mesoporous silica shell due to the capping effect of collapsed PAA brushes, while in pH 7.4 phosphate‐buffered saline (PBS), DOX easily released from the pores due to the brush swelling. PAA‐functionalized UCNP nanohybrids were found to display improved pH‐responsiveness in response while enabling upconversion luminescence‐based bioimaging, resulting in improved therapeutic efficacy (**Figure** **8**A).^[^ ^108^ ^]^

Responsive polymer brushes have also been applied to the design of magnetic resonance imaging (MRI) contrast agents able to deliver therapeutics. For instance, multifunctional magnetic mesoporous silica nanospheres (MMSNs) were decorated with PAA brushes introduced via ATRP of *tert*‐butyl acrylate prior to deprotection.^[^ ^109^ ^]^ DOX was loaded into the complex and subsequently released in a pH‐responsive sustained manner. These multifunctional pH‐responsive MMSN‐PAA nanocomplexes were simultaneously studied as an effective MRI contrast agent and novel drug delivery tools for cancer treatment in vitro (HeLa cells). Yar et al. developed theranostics nanoparticles based on superparamagnetic iron oxide nanoparticles (SPIONs) as the core and the thermoresponsive PNIPAAm shell, generated via ATRP, for the release of DOX in HeLa cells (Figure 8B).^[^ ^110^ ^]^ Release of DOX under physiological conditions was below 20%, but around 90% at 42 °C, allowing to increase drug efficacy mediated by hyperthermia. These multifunctional nanoparticles also reduced the signal intensity significantly in the T2 mode, therefore displaying promising properties as MRI contrast agents. Liu et al. reported the formation of glutathione‐responsive system in which SPIONPs presenting DOX‐loaded PAA brushes formed complexes and were encapsulated inside nanovesicles.^[^ ^111^ ^]^ The obtained nanovesicles responded to changes in glutathione concentration in vivo and lead to drug release and T1 contrast activation. Integrating biomedical imaging and multimodal therapies into one platform is attractive for enhanced anticancer efficacy. Zhang et al. prepared multifunctional structured nanotheranostic with PNIPAAm‐*co*‐PMAA brush shells for MRI‐guided chemo‐photothermal therapy.^[^ ^112^ ^]^ The release of loaded DOX was sped up both via a reduction in pH and increased temperature, induced by NIR light irradiation of the photosensitive CuS cores. These nanomaterials displayed long systemic circulation following intravenous injection to 4T1 tumor‐bearing mice and considerable accumulation at tumor sites, visualized by MRI. This study demonstrates the potential of synergistic therapeutic effects of photothermal and chemotherapy for cancer therapy. In addition to commonly used anticancer drug DOX, a range of other small molecules, including aspirin,^[^ ^127^ ^]^ alizarin red S,^[^ ^101^ ^]^ 4‐acetamidophenol, and ranitidine^[^ ^102^ ^]^ have also been delivered via responsive polymer brush‐based systems (Table 1).

Responsive polymer brush‐based systems for chemotherapy have also been combined with immunotherapies. For example, the pH and redox‐responsive anticancer prodrug poly(SN_38_ (7‐ethyl‐10‐hydroxycamptothecin)‐*co*‐4‐vinylpyridine) was grafted to PEGylated nanogapped gold nanoparticles (AuNNP@PEG/PSN_38_VP). The modified AuNNPs further self‐assembled into a nanocomplexed vesicles and loaded with small molecular immune inhibitor 4‐[[2‐[[(1R,2R)‐2‐hydroxycyclohexyl]amino]‐6‐benzothiazolyl]oxy]‐*N*‐methyl‐2‐pyridinecarboxamide (BLZ‐945) (AuNNP@SN_38_/BLZ‐945) to enable imaging‐guided chemo‐immunotherapy.^[^ ^113^ ^]^ At low pH, BLZ‐945 targeting tumor‐associated macrophages were decomposed by these responsive AuNNP@SN_38_/BLZ‐945 vesicles, followed by deep penetration of AuNNPs@SN_38_ into the tumor. Within the reductive tumor microenvironment, SN_38_ was released to induce cancer cell apoptosis. In the NIR range, AuNNP@SN_38_/BLZ‐945 showed very strong photoacoustic absorbance. Thus, the release of BLZ‐945 can be tracked and mediated by the photoacoustic imaging. Its therapeutic effect can be monitored by varying the photoacoustic signal and intensity. A similar example has been reported by Feng et al.,^[^ ^114^ ^]^ in which binary cooperative prodrug nanoparticle (BCPN) constructed from a dual responsive oxaliplatin prodrug was coated by a reduction‐responsive homodimer of NLG919 (1‐cyclohexyl‐2‐(5*H*‐imidazo[5,1‐a]isoindol‐5‐yl)ethanol, a potent indoleamine 2,3‐dioxygenase (IDO) pathway inhibitor). Upon acidity and reduction in the tumor microenvironment, oxaliplatin and NLG919 were activated to promote intratumoral accumulation of cytotoxic T lymphocytes. In addition, NLG919 was found to downregulate IDO‐1‐mediated immunosuppression and suppressed the activation of regulatory T cells. This synergistic system showed higher tumor suppression efficiency than free oxaliplatin or the combination of free oxaliplatin and NLG919 in both breast and colorectal mouse cancer models.

The SiRNA targeting program death‐ligand 1 (siPD‐L1) was recently reported as an important checkpoint gene for cancer cell survival. SiPD‐L1 was conjugated to magnetic nanoparticles via redox‐responsive *N*‐succinimidyl 3‐[2‐pyridyldithio]‐propionate (SPDP) to achieve MRI‐guided pancreatic cancer immunotherapy.^[^ ^125^ ^]^ Luo et al.^[^ ^126^ ^]^ also reported an MRI‐guided siPD‐L1 delivery system targeting folate receptor which was overexpressed in many cancers. SPIO nanoclusters were grafted with redox‐responsive block copolymers functionalized with folic acid (FA‐PEG‐SS‐PEI, folic acid‐poly(ethylene glycol)‐disulfide‐poly(ethylene imine)). The polyplex exhibited significant enhancement of cellular uptake and T2‐weighted contrast. Additionally, PD‐L1 expression was downregulated at both the mRNA and protein levels, which highlights the potential of these nanosystems in restoring T‐cell immune response in vitro.

Responsive polymer brush‐assisted drug delivery systems are also capable of controlled delivery of larger bioactive molecules, e.g., proteins and enzymes. For example, Yu et al. reported temperature‐controlled delivery of lysozyme, an antibacterial enzyme that plays an important role in targeting Gram positive bacteria.^[^ ^115^ ^]^ In another example, Yavuz et al.^[^ ^116^ ^]^ anchored dense PNIPAAm‐*co*‐PAAm copolymer brushes to Au nanocages, triggering DOX and lysozyme release upon NIR laser irradiation and associated photothermal effects (Figure 7C). Above the LCST, the polymer chains collapsed to open the pores of the cage and release the loaded drugs. The local temperature dropped immediately when the laser was switched off, so the polymer chains relaxed back to form an extended state, which closed the pores of the cage and blocked further drug release. Together with high temporal and spatial resolution, this delivery system is suitable for in vivo studies due to high transparency of soft tissue in the NIR region.

Nucleic acid delivery is another application for which responsive polymer brushes (e.g., cationic polymer brushes) have attracted attentions. A number of responsive polymer brush‐coated nanoparticles were designed to deliver plasmid DNA for potential cancer therapy and combined treatments, mainly based on PDMAEMA brushes (Table 1). Interactions between DNA/RNA molecules and polymer brushes have been systematically investigated for a better understanding of the nature of formed complexes. The molecular environment including type of buffer, pH, and concentration on the interactions between positively charged pH‐responsive PDMAEMA brushes and plasmid DNA were examined using in situ ellipsometry, light scattering, surface plasmon resonance, etc.^[^ ^36^ ^]^ The conformation of swollen brushes was found to be modulated by the type of buffer used, impacting strongly on the ability of such brush‐coated nanomaterials to complex DNA molecules. Transfection efficiencies correlated with such changes in brush conformation and DNA binding were found to be significantly higher when complexes were formed in PBS and NaCl solution than in 4‐(2‐hydroxyethyl)‐1‐piperazineethanesulfonic acid buffer. The molecular design of polymer brushes was found to play an important role in controlling DNA complexation and release in a triggered drug delivery system. This was supported by an adsorption model that combined a simple polyelectrolyte surface adsorption model with an infiltration step dependent on brush architecture.^[^ ^95^ ^]^ The brush grafting density and thickness were found to greatly impact on the absorption profile of oligonucleotides. For instance, the small oligonucleotides (10 bp) infiltrated relatively fast into the brushes, with a relatively low binding affinity and moderate binding factor (and loading level). Interestingly, the charge balance of complexes formed between RNA (20 bp) and PDMAEMA brushes was found to be close to unity, indicating a particularly tight and stable binding. In contrast, DNA oligonucleotides of similar sizes displayed weaker interactions and stabilized with an excess positive charge. This model also speculated that the design of responsive polymer brushes with more dense and complex structures, such as block copolymers, may further alter the adsorption dynamics and release kinetics of the associated vectors, potentially allowing to confer additional properties (such as reduced cytotoxicity). Interestingly, highly structured block copolymer brush‐coated nanoparticles were reported to show the protection of oligonucleotides by a protein‐resistant outer block (Figure 7D).^[^ ^18^ ^]^ Such brush hybrids retained excellent transfection efficiencies, while displaying an improved protein resistance, high serum stability, and low cytotoxicity.

Overall, responsive polymer brush‐functionalized nanomaterials are promising gene delivery systems and can conveniently be combined to a variety of cores with imaging capabilities, such as fluorescence or MRI, due to the simplicity with which brushes (e.g., PDMAEMA) can be grown from a range of nanomaterial cores. This was demonstrated in the case of nanodiamond cores grafted with cationic PDMAEMA via ATRP for enhanced plasmid DNA delivery and bioimaging using the inherent fluorescence of nanodiamond.^[^ ^128^ ^]^ Similarly, upconversion nanoparticles conjugated with photo‐induced and charge‐variable polyelectrolyte brushes allowed simultaneous control of siRNA release and photodynamic therapy under irradiated NIR light (Figure 8C).^[^ ^124^ ^]^ In addition, macroinitiators can be combined with fluorescent polyeletrolyte conjugated polymers to enable imaging of the distribution of nanomaterial vectors within the cytoplasm.^[^ ^94^, ^126^ ^]^ To further improve the performance of responsive polymer brush‐based vectors for gene delivery, some challenges remain. Although plasmid DNA and RNA (siRNA and miRNA) delivery typically only requires very low loading levels (a few copies per cell), the loading capacity of these systems could be improved to reduce the number of vectors required for delivery. In addition, the mechanism of release of RNA/DNA from polyplexes, including those based on responsive polymer brushes, remains poorly understood, yet may allow refinement of the design of associated vectors. Such improved design may also be applied to the delivery of other therapeutic cargos such as enzymes. Finally, as described in Table 1, most of the work published so far remains focused on in vitro delivery and translation of these systems to in vivo delivery appears promising but remains to be studied.

### 3.2 Responsive Polymer Brushes in Biosensing and Detection

Polymer brushes can be used as sensing elements for biodetection. There are increasing reports on the combination of responsive polymer brushes with nanomaterials for smart sensing in disease diagnosis. Stimuli‐responsive polymer brushes are capable of responding to the physical, chemical, and biological stimuli from external analytes and convert such information into physicochemical changes. These systems facilitate efficient transduction mechanisms which are well suited for use in biosensing. Among applications pertaining to this, responsive polymer brush‐based nanosensors (**Table** **2**) have attracted considerable attentions for the selective recognition and quantification of biological factors such as levels of glucose, biomarkers, enzyme, and bacteria.

**Table 2:** A summary of recent applications of responsive polymer brush nanomaterials applied to biosensing

Factor^a)^	Stimuli	Responsive polymer brush	Sensing method	Sensitivity achieved	Ref.
Temperature	Temperature	PNIPAAm	LSRP of AuNPs	The O.D. of absorption increases from 0.267 to 0.291 with temperatures ranging 20–40 °C	^[^ ^129^ ^]^
pH	pH	PNIPAAm or (PNIPAAm‐*co*‐PVI)	Micro‐cantilevers	Sensitivity of 121 nm/pH unit (pH range: 4–6)	^[^ ^130^ ^]^
pH	pH	PMEP	Cantilevers	Max surface stress: 3 N m^−1^	^[^ ^131^ ^]^
pH	pH	P2VP	AuNPs‐enhanced SPR	50 nm shift in absorption at pH 2–5	^[^ ^132^ ^]^
pH	pH	PDMAEMA	LSPR of AuNPs	Absorption band shift of 10 nm in pH range 5–9	^[^ ^133^ ^]^
Organic solvent	Solvent	PS	LSPR of AuNPs	32 nm blueshift upon change of solvent	^[^ ^134^ ^]^
Cationic redox species	pH	P2VP and PAA mixed brush	Electrochemistry	Potential shift based on pH (46 mV per pH)	^[^ ^135^ ^]^
Glucose	pH and glucose	PNIPAAm‐*co*‐PAA‐PBA	Micro‐cantilevers	max surface stress change: 1.69 N m^−1^	^[^ ^136^ ^]^
Glucose	pH	P4VP	Electrochemistry	Potential change of 67 mV per pH within pH range of 3–7	^[^ ^137^ ^]^
Glucose	pH	P2VP	Electrochemistry	Dynamic concentration range of 2–16 mmol L^−1^ LOD of 5.6 × 10^−6^ m	^[^ ^138^ ^]^
Glucose	Glucose	PMAPBA	QCM‐D	Shift in resonance frequency (0–30 Hz) over [glucose] in range of 0–100 × 10^−3^ m	^[^ ^139^ ^]^
Glucose	pH and temperature	P2VP, PAA, or PNIPAAm and PAA mixed polymer brush	Spectroscopic ellipsometry and ATR‐FTIR	Thickness change (90–20 nm) at temperatures from 20 to 40 °C	^[^ ^13^ ^]^
*γ*‐IgG	Mass	PLL‐PEG‐biotin	Fluid‐filled micro‐cantilever	Sub‐femtogram resolution (sub‐monolayer)	^[^ ^140^ ^]^
Protease biomarker (trypsin, MMP‐2, MMP‐9)	Peptide as the substrate of enzyme	PMAA	Fluorescence microscopy	LOD of 1.8 × 10^−12^ m	^[^ ^141^ ^]^
*Streptococcus mutans*	Temperature	PNIPAAm	Confocal microscopy	Cell densities of 0.6–1.2 × 10^6^ cfu cm^−2^ (temperatures 4–37 °C)	^[^ ^142^ ^]^
Osteosarcomic Soas‐2 cells	Temperature	PNIPAAm	Silicon nanowire‐based FET	Sensitivity of ≈59 mV °C^−1^ 0.15 V potential shift within pH change of 5.6–8.0.	^[^ ^143^ ^]^
	pH and ionic strength	PAA			

^a)^ Abbreviations: *γ*‐IgG, immunoglobulin G; PNIPAAm, poly(*N*‐isopropylacrylamide); PNIPAAm‐*co*‐PVI, poly(*N*‐isopropylacrylamide‐*co*‐*N*‐vinylimidazole); PMEP, polymethacryloyl ethylene phosphate; P2VP, poly(2‐vinylpyridine); PDMAEMA, poly(*N*,*N*′‐dimethylaminoethyl methacrylate); PS, polystyrene; PAA, poly(acrylic acid); PNIPAAm‐*co*‐PAA‐PBA, poly(*N*‐isopropylacrylamide)‐*co*‐poly(acrylic acid)‐(3‐aminophenyl‐boronic acid); P4VP, poly(4‐vinyl pyridine); PMAPBA, poly(3‐methacrylamido phenylboronic acid); PLL‐PEG‐biotin, poly(ethyleneglycol‐biotin)‐graft‐poly(L‐lysine); PMAA, poly(methacrylic acid); PAA, poly(acrylic acid); LSPR, localized surface plasmon resonance; AuNPs, gold nanoparticles; SPR, surface plasmon resonance; QCM‐D, quartz crystal microbalance with dissipation monitoring; ATR‐FTIR, attenuated total reflection‐Fourier transform infrared spectroscopy; FET, field effect transistors; O.D., optical density; LOD, limit of detection.

**Figure 9:**
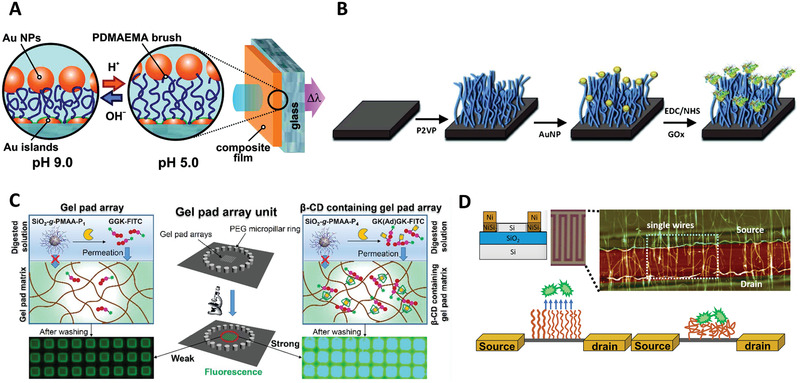
Examples of responsive polymer brushes for biosensing and diagnosis. A) Optical plasmonic nanosensor platform based on PDMAEMA brushes operating at near‐physiological pH. Reproduced with permission.^[^ ^133^ ^]^ Copyright 2011, American Chemical Society. B) Stimuli‐responsive biointerface based on polymer brushes for glucose detection. Reproduced with permission.^[^ ^138^ ^]^ Copyright 2014, Wiley. C) Peptide‐functionalized PMAA brushes for on‐chip detection of protease biomarkers. Reproduced with permission.^[^ ^141^ ^]^ Copyright 2018, American Chemical Society. D) Schematic representation of PNIPAAm brushes immobilized on SiNW FETs for biosensing. Reproduced with permission.^[^ ^142^ ^]^ Copyright 2005, The Royal Society of Chemistry.

Among various responsive polymer brushes, pH sensitive brushes are commonly used for detection. For example, polyelectrolytes poly(2‐vinyl pyridine) (P2VP) and PAA brushes were prepared from indium tin oxide (ITO) conducting glass–electrode surfaces^[^ ^135^ ^]^ coated with primary silane layers. The developed electrochemical nano‐transistor allowed reversible and selective reactions of cationic and anionic redox species (i.e., [Fe(CN)_6_]^4−^, [Ru(NH_3_)_6_]^3+^) at different pH. These redox species are useful as electron‐transfer mediators between redox enzymes and the switchable electrode interface, as well as in many bio‐electrocatalytic sensing systems. This system constituted a model to mimic the electrochemically induced penetration of cationic drugs. An optical nanosensor working at near physiological pH condition was reported based on PDMAEMA brush modulated‐plasmon sensing (**Figure** **9**A).^[^ ^133^ ^]^ This pH sensor exploited the combination of the swelling–shrinking transition in a PDMAEMA brushes and the localized surface plasmon resonance in noble metal nanoparticle composites to transduce pH signal in the solution from 5.0 to 9.0 into a pronounced optical signal. The sensor is advantageous to measure the solution pH within near‐physiological range, which makes it possible to merge with many biomedical diagnostic tools such as enzymatic reactions. Many other pH‐responsive polymer brush systems have also been reported so far.^[^ ^130^, ^131^, ^132^, ^144^ ^]^ Apart from these examples, responsive polymer brush‐coated nanomaterials are also used for detection of temperature change,^[^ ^129^ ^]^ electrical potential change,^[^ ^145^ ^]^ and organic solvents.^[^ ^134^ ^]^

Glucose is one of the most popular target analytes by responsive polymer brush‐based sensors. Stimuli‐responsive biointerfaces based on polymer brushes have been reported for glucose detection, such as a poly(3‐methacrylamido phenylboronic acid) (PMAPBA) brush‐modified quartz crystal microbalance with dissipation monitoring (QCM‐D) sensor.^[^ ^139^ ^]^ This PMAPBA brush‐modified QCM‐D sensor responds to a range of physiologically relevant glucose concentrations (5–100 × 10^−3^ m) and the bio‐detection was specific as not significantly disturbed by competitive binding of fructose. Although the relatively high p*K*a value of the PBA moiety (≈8.8) may not allow direct analysis of serum samples or glucose monitoring in vivo, such glucose‐sensitive QCM‐D sensor can be used for in vitro diagnostic purposes or point‐of‐care testing. Crulhas^[^ ^138^ ^]^ et al. reported a stimuli‐responsive glucose biosensor based on P2VP brushes with glucose oxidase (GOx) as an enzymatic probe for blood test (Figure 9B). P2VP brushes change their conformation in response to pH due to the oxidation of glucose in the sample solution. In turn, the different states (swollen at low pH or shrunken at high pH) of brushes influence the diffusion of redox active compounds to the electrode surface. At low pH, the P2VP brushes enable transfer of electrons more efficiently, resulting in a more sensitive glucose biosensor with a dynamic concentration range of 2.0 × 10^−3^–16.0 × 10^−3^ m. Another similar study focused on pH‐switchable bioelectrocatalytic oxidation of glucose modulating the conformation of P4VP brushes in the presence of soluble GOx.^[^ ^137^ ^]^ This glucose biosensor responds to changes in pH between 4.0 and 7.0, resulting in the reversible switching of the bioelectrocatalytic process. This pH‐dependent behavior arises from the restructuring of P4VP polymer brushes and associated switch between the electrochemically active or inactive states for electron transport. For such responsive polymer brush‐mediated glucose detection, the adsorbed amount and the catalytic activity of the immobilized GOx are essential to the detection performance. Koenig^[^ ^13^ ^]^ and co‐authors investigated the influence of pH on the amount and activity of GOx enzymes adsorbed to P2VP or PAA brushes, as well as the creation of thermo‐responsive biocatalytical coatings via the adsorption of enzymes onto a mixed brush consisting of P2VP or PAA with PNIPAAm brushes, respectively. PAA brushes generally adsorbed larger amounts of enzyme, while reduced GOx were observed at the surface of P2VP brushes. However, the latter brushes exhibited higher specific activity. In the case of GOx adsorbed to mixed brushes, switching of enzymatic activity between an active state at 20 °C and a reduced state at 40 °C was observed. This work suggests that the conformation and design of responsive polymer brush‐based sensors play important roles in achieving efficient biocatalytic efficacy.

In addition to the detection of small biological molecules, biomacromolecules including antibodies and protease biomarkers can also be sensitively detected using responsive polymer brush‐based biosensors. Due to the ionization of carboxyl groups, PMAA brushes are highly susceptible to swelling in aqueous solutions, resulting in marked pH and ionic strength sensitivity. The abundance of carboxyl groups on PMAA brushes also allows versatile conjugation strategies for the immobilization of functional molecules. Hence, PMAA brushes‐grafted silica nanoparticles have been used for the detection protease biomarkers using a polyacrylamide (PAAm) gel pad array chip (Figure 9C). The conjugated peptides on PMAA brushes were used as substrates for relevant proteases such as trypsin.^[^ ^141^ ^]^ To realize point‐of‐care detection of proteases, PAAm gel pad arrays were used for permeation of fluorescein‐labeled peptide fragments cleaved from the PMAA brushes and quantified by fluorescence microscopy. This on‐chip protease assay detected trypsin with a limit of detection (LOD) of 3.9 × 10^−12^ m (buffer solution) and 1.4 × 10^−9^ m (serum), with good specificity toward chymotrypsin. Given recent advances in the simplification of sample handling, such rational design of peptide‐functionalized PMAA brushes could also be used for the screening of protease inhibitors. The protein detection sensitivity using responsive polymer brush‐based biosensors can be further enhanced.^[^ ^15^ ^]^ For instance, Burg^[^ ^140^ ^]^ and co‐workers proposed a highly sensitive (sub‐fg level) detection of immunoglobulin G (*γ*‐IgG) using poly‐L‐lysine‐PEO‐biotin brushes grafted inside a confined microchannel carved within a microcantilever. This microcantilever sensor responded selectively and sensitively upon the binding of *γ*‐IgG, representing a significant improvement in sensitivity over conventional quartz microbalance.

Stimuli‐responsive polymer brushes have also been applied for the sensing of tumor cells or bacterial pathogens. Alarcón et al.^[^ ^142^ ^]^ demonstrated the use of micropatterned PNIPAAm brushes on gold surfaces for the investigation of interactions of a specific bacterial strain, *Streptococcus mutans* (Figure 9D). The adsorption of this common oral bacterium varied with temperature, following “cycling” of the PNIPAAm brushes above and below the LCST. In another similar example, PNIPAAm brushes were functionalized on silicon nanowire‐based field effect transistors (SiNW FETs) for the detection of human osteosarcomic Saos‐2 cells.^[^ ^143^ ^]^ PNIPAAm brushes encountered reversible conformational changes in exposure to cell‐mediated external stimuli and monitored via the FET response. The use of this mixed polymer brush system could open up possibilities for designing highly reversible and tuneable biosensing systems for the detection of a variety of biological analytes including tumor cells.

Overall, although responsive polymer brushes have not been directly used for systematic studies for theranostic purposes, they have shown great potentials to be used as sensing platforms for the detection of biological analytes. However, challenges remain to see their wider application in nanotheranostics. For instance, highly sensitive responsive polymer brush‐based biosensors have to be built together with efficient signal readout mechanisms in order to sense the target molecules at physiologically relevant concentrations. Selective responsive brush structures would contribute largely to specificity in detection and could rapidly be adapted to a broader range of biomarkers.

## 4 Summary and Perspectives

In summary, we presented the recent advances in the rational design and synthesis of responsive polymer brushes for applications in smart delivery systems and biosensors/detection. Among other polymeric platforms for theranostic applications, responsive polymer brush‐based systems exhibited unique advantages for improved control over the responsiveness and physiochemical properties of the polymer coatings, essential to the control of interfacial interactions between associated nanomaterials and their biological environment. The versatility and precision in design and fabrication of responsive polymer brush‐based materials constitute a clear advantage for application in the biomedical field, as intelligent delivery platforms for therapeutic molecules and smart biosensing/detection tools. The translation of these systems to the field of nanotheranostic remains limited but can now progress fast, as the toolbox of cores and responsive polymer brushes available to bioengineers has rapidly extended in the last two decades. However, key challenges remain to be addressed to enable these smart coatings to be used more widely and to improve our understanding of mechanisms regulating the response of associated nanomaterials in a physiological environment in vivo. First, the development of more versatile and bio‐friendly synthesis methods of responsive polymer brushes is needed. Polymerization methods that involve the use of metal catalysts may add extra purification steps to completely remove associated metals, which may constitute a regulatory hurdle for clinical applications. The responsive polymer brush growth from different cores dependent on the surface chemistry and the design of suitable initiating moieties can be further refined. Thus, more widely applicable approaches should be developed to offer more freedom to the brush fabrication from different nanomaterials. Second, a more comprehensive insight into the interactions between biological systems (proteins, oligonucleotides, phospholipid membranes, and other small molecules physiologically abundant) and brush coatings is still required. This also includes the investigation of interactions between brushes and complex biological systems such as complex physiological fluids, cells, bacteria, and tissues. Finally, for the application of biosensing and bioresponsive polymer brushes for nanotheranostics, the incorporation of efficient signal readout with sensitive polymer brush systems coupled to nanomaterials conferring imaging or acting as reservoirs for drug release largely remains to be developed. The potential is clear though and responsive polymer brushes offer the opportunity to combine highly sensitive and specific detection of disease biomarkers with delivery and imaging platforms.

## Conflict of Interest

The authors declare no conflict of interest.
